# Bridge Non-Destructive Measurements Using a Laser Scanning during Acceptance Testing: Case Study

**DOI:** 10.3390/ma15238533

**Published:** 2022-11-30

**Authors:** Pawel Tysiac, Mikolaj Miskiewicz, Dawid Bruski

**Affiliations:** EKO-TECH Centre, Faculty of Civil and Environmental Engineering, Gdansk University of Technology, 80-233 Gdansk, Poland

**Keywords:** bridge load tests, terrestrial laser scanning, structural health monitoring, deformation analysis, non-destructive testing, maintenance

## Abstract

Owing to the recent proliferation of inventory works on roads and railways, bridge acceptance tests have increased exponentially. These tests’ results are often misinterpreted owing to the use of various measuring equipment types, rendering integrated interpretation problematic. It is also problematic that adjusting the measurement method is difficult when the structure’s response to load is uncertain. Therefore, it is important to observe the largest possible range of possible deformations. For this reason, the present study suggests a novel approach to bridge non-destructive measurements using a laser scanner during acceptance testing. The main advantage of our method is the ability it affords to observe all points of the structure during testing, an ability that is extremely important is the absence of unambiguous data regarding the bridge’s condition. To significantly increase the scanning accuracy (up to 0.5 mm), measurements from a limited number of linear sensors are used (whose accuracy is up to 0.1 mm). To achieve optimal accuracy, we performed the following steps: first, we adapted the precision requirements to the numerical project. For this purpose, we used potentiometric sensors to measure linear deformations. Next, we performed laser scanning measurements from two scan positions. Finally, we filtered the data for the selected cross-section and modelled the points into polynomial deflection. The performed tests confirmed that the structure’s response was as predicted by the FEM model, and the object was approved for use. Our future tests will be based on the selection of a structure with minimal measurement errors, and the results will be compared using a total station, ensuring the highest possible quality of service, which can be repeated in simple steps. As study objects, we presented two items: the first without proper calibration on a linear sensor and the second using linear sensors to present the highest possible accuracy of our experiment.

## 1. Introduction

### 1.1. Problem Description

Research on bridge structures can be broadly divided into two ranges. The first range comprises acceptance tests [1,2], the primary goal of which is to verify the structure’s geometrical response to the influence of load based on a calculation model. The second range concerns the diagnosis of existing objects [3,4]. Test loads are particularly valuable in the case of unusual, innovative constructions and material solutions, providing information that may confirm the theoretical analysis or reveal minimal discrepancies. Moreover, the use of measuring instruments, such as laser scanners, total robotic stations, or potentiometric sensors, may not reflect the object’s actual geometrical situation when applied and interpreted individually. Laser scanners record spatial data with millimetre-level accuracy that increases with each new position alignment [5,6,7,8,9]. A total robotic station can measure below the millimetre level, but its key role is to arrange the prisms on the structure [10,11]. Thus, the values between the prisms are usually approximated. Potentiometric sensors provide information at installation sites based on a single altitude coordinate. As a novel application, the paper presents the use of a laser scanner together with sensors used to measure linear deformations supported by finite element method (FEM) calculations [6]. Such a solution is of the utmost salience in the case of non-standard bridge constructions and those that have suffered a failure. Despite the use of the FEM model, there can be no guarantee that the structure will react as predicted under load. For this reason, it is crucial to observe as many points on the structure as possible with the greatest possible accuracy. The solution that we propose provides such an opportunity while simultaneously reducing the costs associated with the time-consuming and costly assembly of prisms and cables used in other measurement systems. Based on measurements from only a few reference points, the proposed solution permits highly accurate observation of the entire structure’s deformations. Additionally, the tests were performed under unfavourable weather conditions (i.e., no daylight and high air humidity). In this way, we wished to highlight the method’s versatility, its advantage over optical systems that need light (based on images) and its reduction of the time taken to place sensors on the structure. In connection with the above, deformation results were presented from the locations specified by the project, where no spatial gaps exist between points on the structure. In this research, the deflection polynomial is presented in the direction of interpolation and not an approximation, thus enhancing the study’s precision and accuracy. In sum, we meet the scientific market’s expectations with respect to the appropriate adjustment of measurement methods to the numerical model and their appropriate interpretation and use for non-standard bridge structures.

### 1.2. Laser Scanning System

Recent developments in laser scanning technology prompted us to adopt a terrestrial laser system (TLS) in our research as the main technology that meets the requirements of precision and accuracy at the millimetre level [12,13,14]. In construction types for which we are unsure of the structure’s response under load, such accuracy may not be sufficient. As such, we knew that, to ensure the highest possible accuracy of the tests, the point cloud should be properly calibrated. In this way, we were able to observe several points on the structure without approximating their location, providing certainty about the bridge’s response under load influence. Manufacturers are surpassing one another in constructing devices capable of collecting millions of points per second with high precision and accuracy (at the level of approximately 1 mm per 10 m) in various scientific research fields [15]. Moreover, the range of wavelength emitted by the device is also applicable in other fields, such as cultural heritage [16] or building inspection [17,18,19,20]. Therefore, a niche for terrestrial scanning technology was found—namely, observation of the continuity of recorded points below the accuracy specified by the manufacturer. However, there are a few requirements that the device must meet for the experiment to succeed. For example, a specific wavelength of light must be less sensitive to the weather conditions at the site, and a longer wavelength would allow for such a possibility [21,22,23,24,25,26,27,28,29,30,31]. In turn, water absorbs red and near-infrared light (most commonly used in instruments) most strongly. Therefore, we formulated a thesis that the most important factor determining the model’s accuracy for further processing is the precision of its acquisition and its ability to reduce the noise produced by the instrument. Acknowledging these possibilities, the appropriate instrument was selected for the research (Leica P30 Manufacturer: Leica Geosystems AG, part of Hexagon, Zurich, Switzerland), taking into account the accuracy and precision of the obtained data. Moreover, the final model described in this paper is characterised by increased accuracy in testing bridge structures. The selected object for the study presented in this manuscript is a bridge with an unusual structure (its upper layers were struck during construction). It was selected because, in typical constructions, the result presented in this paper may not be unique in comparison to other known measurement methodologies [29,30,31].

As a rule, bridge load tests last several hours and are influenced by numerous logistical factors (e.g., stacking trucks on the span). Moreover, the tests are planned many weeks in advance, involving considerable material and human resources. Therefore, the measurement system must be reliable by design, ensuring that it can be measured regardless of the time of day. Moreover, it must be as resistant as possible to unfavourable weather conditions. For the reasons described above, despite the possibility of obtaining greater accuracy, digital image correlation (DIC) [32] methods (for example) may not be sufficient in light of the relatively small area of observation and the significant influence on lighting conditions. In addition, measurement using a laser scanner is based on non-invasive mirrorless measurement. This solution involves no obligation to install additional sensors on the structure (e.g., in the form of fiber-Bragg-grating-based sensors) [33]. The calibrated points from the scanner at a distance of no more than a few centimetres can be observed separately without approximating the results between these points. The summary of the results and their presentation will shed light on the practicality of our proposed method, which assumes the use of other sensors for point cloud calibration rather than for comparative analysis. This is a significant methodological change. An earlier study [34] employed a construction similar to the numerical analysis but whose results were inaccurate compared to the total station. This solution emerged as a result of differences in the interpretation of norms relating to now-outdated approaches to presenting displacement results [35,36].

Studies concerning the behaviour of newly created bridge structures are often concerned primarily with the presentation of results [37]. Therefore, while issues pertaining to numerical modelling have advanced considerably, the question of how measurement methods might be refined remains largely unresolved [38,39]. Therefore, despite obtaining relatively good results from the FEM model, measurement methodologies have limitations that we have endeavoured to minimise using laser scanning technology, as described above. In order to convince those who use bridges (i.e., people (footbridges), cars (road bridges), or trains (railway bridges)) of their safety, these structures’ theoretical behaviour must be demonstrated. Their fitness for purpose is verified in the field by a sequence of tests [40]. During the investment process, numerical calculations are applied as a standard to determine the structure’s predicted behaviour and facilitate the interpretation of the results [41,42,43,44,45]. The references predict bridge displacements, their accelerations depending on the circular motion of the bridge, and assessment based on their complicated structures. The FEM model’s correctness is verified during field tests, and it is thus crucial that the selection of these methods be approached correctly. Therefore, in addition to reliability, the measurement system must meet the requirements associated with checking the FEM model, which, in our case, was determined by presenting the calculation methodology.

Based on the research that has already been carried out internationally, it can be concluded that in the case of uncomplicated objects, numerical modelling almost always yields the same results as measurements do. The problems arise in relation to non-standard objects—that is, those that have applied innovative technological solutions during construction or where significant changes have been made to the structure in response to various factors. For this reason, two research cases have been performed, one without the use of the methodology described in the article and the other with the use of our methodology. In this way, we demonstrated the full spectrum of the use of laser scanning technology in response to the possibility of ensuring the reliability of the measurement system in relation to other methods (e.g., DIC), thus demonstrating a new approach to bridge operation under load influence. Laser scanning processed data meets the requirements for test loads. To comprehensively present the problems associated with testing non-standard engineering structures, we first present a description of the structures, followed by the non-standard nature of the engineering facility, their numerical calculation methods and, finally, the method of measurement used.

## 2. Materials and Methods

### 2.1. Study Area

**Figure 1:**
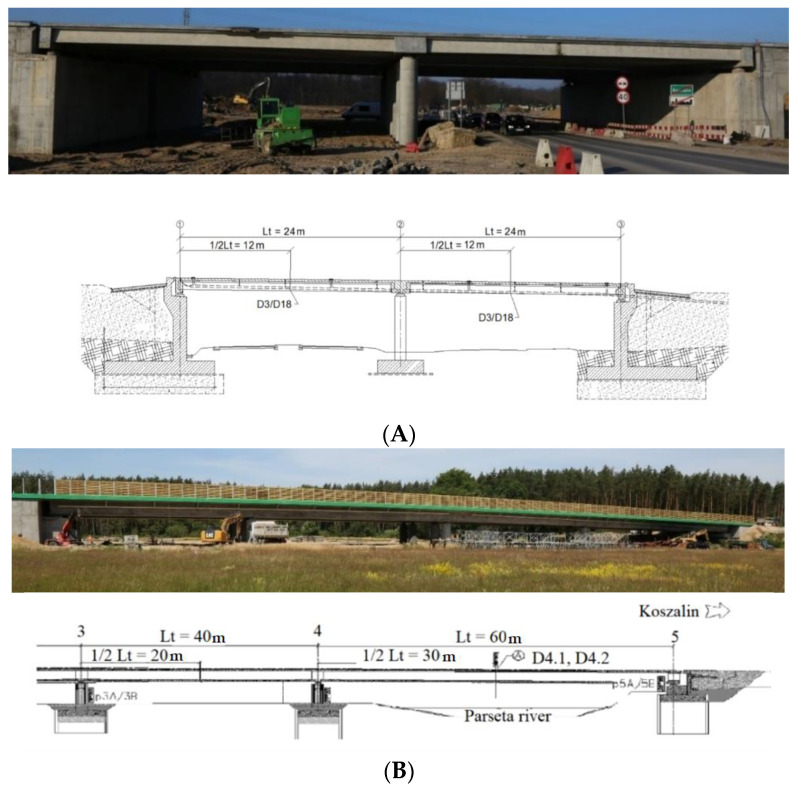
Side view of the bridge with its cross- section (**A**) WD-113, (**B**) M78.

As research objects, the viaduct (WD-113) located along a newly built road next to the city of Koszalin in Poland (Figure 1A) and the bridge (M78) located along a newly built road next to the city of Kolobrzeg in Poland (Figure 1B) were chosen. The viaduct WD-113, constructed from pre-stressed concrete, has a continuous two-span scheme. The building’s load class was designed in accordance with the Polish standard for bridge structures PN-85/S-10030. The cross-section for each span comprises 20 pieces (for the left span) and 20 pieces (for the right span) of prefabricated pre-tensioned concrete beams of the “T” type with a spacing from 0.90 to 0.95 m. The width of the beams’ upper flange is constant and amounts to 0.89 m. The beams are 0.75 m in height and 17.55 m in length, combined with a reinforced concrete platform slab of 24 cm thickness. The superstructure’s structural height is 0.99 m. Reinforced concrete crossbars have been designed above the abutments and intermediate supports. The crossbeams above the abutments are 1.49 m high, while the crossbeams above the intermediate supports are 1.23 m. Pavement covers are concreted onto the supports on which prefabricated cornice elements made of polyester-glass laminate, as well as protective barriers and balustrades, are also mounted. The supporting beams are mounted on supports by means of pot bearings. The load-bearing structure of each roadway of the M78 bridge was made in the form of a four-span continuous beam of pre-stressed concrete with a single-chamber box section. The cross-section height is constant and amounts to 2.65 m. In the cross-section, the box consists of inclined webs of variable thickness (min. 45 cm), monolithically connected with a bottom plate of variable thickness (min. 25 cm) and with an upper plate, also of variable thickness (min. 30 cm). A 2.755 m long cantilever, symmetrical in relation to the main axis of the object, was led out of the box. The thickness of the brackets is variable-from 21 cm at the end of the bracket to 50 cm at the place of its mounting (in the web).

The non-standardised nature of the viaduct WD-113 derived from the damage that it incurred from the impact of a rolling stock truck with the pre-stressed precast concrete T-beam girders. As a result of the collision, the concrete was chipped on the girder edge, and numerous scratches occurred. The repair of the damaged structure entailed combining the extreme and pre-boundary girders to ensure that they would cooperate when transferring moving loads. In the case of the M78 bridge, our objective was to demonstrate the principles of measurement without using the methods presented in this article and without achieving satisfactory results. It allows us to understand the meaning of the article and to make more popular the idea of our research on other bridge structures.

### 2.2. Standard Scope of Tests for Object’s Non-Standard Nature (M78)

**Figure 2:**
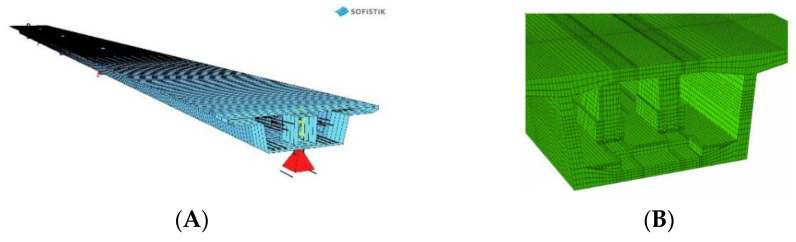
Representation of the M78 FEM model: (**A**) Global (SOFiSTiK), (**B**) Local (ABAQUS).

In the case of the M78 bridge, the visual inspection of the structure was carried out before the test load, and it confirmed that the concrete of the extreme supporting crossbars was cracked. Moreover, it was observed that on selected scratches, gypsum and glass fillings had been previously placed. These seals were damaged (broken) before the tests were started. In the bottom plate, in the area of the anchor block for which the repair program was carried out, a loss of concrete cover from the outside of the box girder was found. There were no other irregularities, including those concerning the bearings, that could affect the course of the test load. No wearing course (SMA) has been noted. After the visual inspection, the object was approved for a load test. The scope of the test was as follows: measurements of span displacements under static and dynamic loads, settlement of supports (static) and acceleration of the structure point (dynamic). The displacements of the spans were measured with the use of SYLVAC digital sensors, and potentiometric linear displacements with a measuring range of 0 ÷ 50 mm and an elementary plot of 0.01 mm, subsidence of supports were measured with optical precision levels Carl Zeiss Ni005 with a reading accuracy of 0.1 mm. Measurement uncertainties resulting from the accuracy of the measuring apparatus and environmental conditions were determined with the coverage factor k = 1.65 for the confidence level *p* = 95%. For the measurement with precision levelling, the measurement uncertainty is ±0.1 mm. Calculations of the structure response during the implementation of the test load patterns were carried out in two stages. In the first stage, the structure was analysed globally, and for this purpose, the SOFiSTiK software was used. As part of the extended scope, the ABAQUS system was used in the second stage for the local analysis of structure responses. The global model of the M78 object was mapped using one-dimensional, two-node spatial beam Timoshenko-type finite elements. Shear effect and the eccentricity of the beam axis were taken into account, and 483 nodes and 402 elements were used to build the model. For the calculation of internal forces and deflections, material characteristics were adopted in accordance with the executive documentation. In order to create a local model in the ABAQUS program, solid incompatible first-order finite elements C3D8I with improved bending properties were used. Finite element meshes with different geometry variants within the analysed span were used. They contained 372,000–450,000 finite elements. The calculations were performed in the linear range. The boundary conditions of the local model were adjusted to fully reflect the global behaviour of the bridge fragment. The visualisation of the local FEM model is presented in Figure 2.

**Figure 3:**
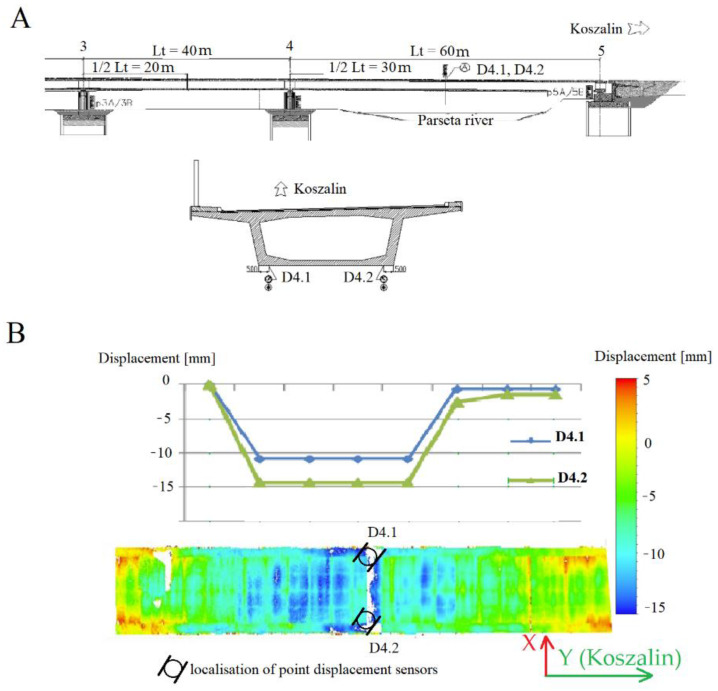
Localisation of potentiometric sensors placement on the M78 bridge (**A**) and Comparison of the test load results of the laser scanner to the potentiometric sensors (**B**).

One of the cross sections of the bridge (marked in Figure 3A as 45), on which vertical sensors were placed and from which laser scanning measurements were performed, was selected for the tests. The results are shown as graphs and deformation maps in Figure 3B. The maximum values of elastic displacements after loads are on the level of 13.05 mm ± 0.1 mm. The theoretical value at this point was equal to 14.40 mm. It means that the measured displacement equals 91% of the theoretical value. Therefore, the average elastic displacement of spans is approximately at level of 86% of the theoretical values. The measured deflections for individual measuring points prove that the transverse and torsional stiffness is correctly assumed numerically. The values of permanent displacements amount to a maximum of 1.40 mm ± 0.1 mm and do not exceed the permissible values of 10% of total displacements. The laser scanning technology performed during the implementation of the test load diagrams confirmed the correct spatial operation of the structure, and the recorded values of the maximum displacements are consistent with the measured precision. Figure 3 shows the results of the bridge load in the form of a TLS deformation map and the graph between the deflection values obtained from potentiometric sensors. From a visual point of view, the maximum deviation value is similar in both cases and indicates the correct functioning of the structure; however, due to the adopted calculation model related to the continuous operation of the scanner and the approximation of the reference surface, there were discontinuities that do not faithfully reflect the work of the bridge structure. It means that there is a need to process the laser scanner data while increasing its reading accuracy. In this way, we motivate the importance of our research. We showed each step leading to the identification of a suitable point model, comparing the function of the deflections that can be approved during the bridge acceptance process. In order to present the universality of the method, the analysis was performed for the WD-113 bridge, which was also of a complex structure. Despite the fact that FEM models should be treated individually depending on the execution method of the bridge structure, such a verifying measurement method can be used independently.

### 2.3. The Object’s Non-Standard Nature (WD-113)

The viaduct’s non-standard nature derived from the truck’s impact with one of its external girders [46]. Geometric inspection of the structure’s technical condition was the motivation for determining the appropriate repair quality and the numerical model. The repairs were executed based on the experience of engineering experts. Consequently, the structure’s behaviour was questionable. In addition, several girders were deformed as a result of the impact. Therefore, the aim of the study was to develop a 3D point model using the active remote sensing method—that is, the use of laser scanning to determine the structure’s geometric deformation in its initial state after the hit. On the basis of the constructed spatial model, the girders were analysed in terms of their geometric correctness from the side of the object under which the movement began and where the direct impact with the structure occurred. The above-mentioned technical analyses are necessary to understand the nature of the bridge itself.

**Figure 4:**
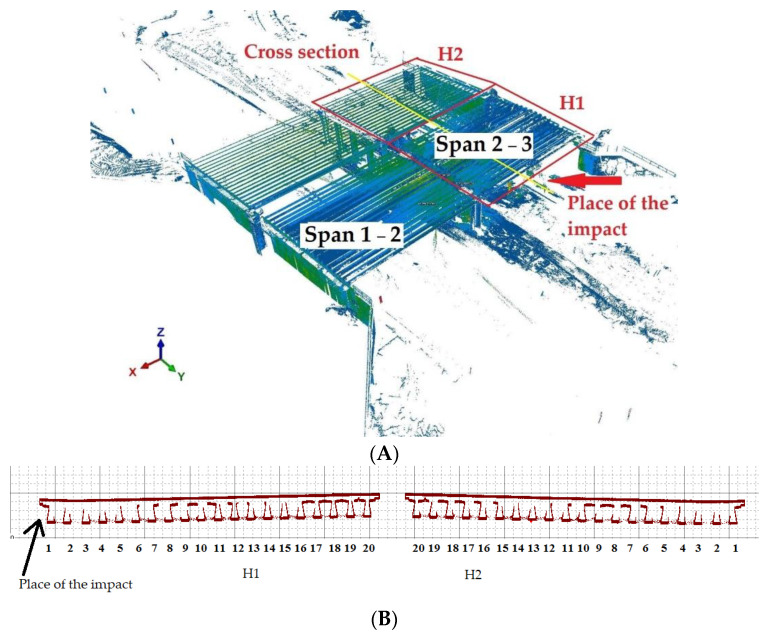
General view of the (**A**) obtained point cloud and (**B**) its cross-section.

**Figure 5:**
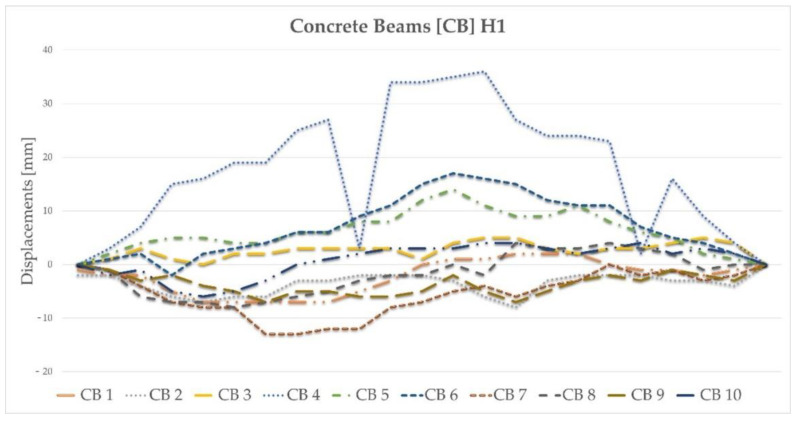
The result of changes in the geometry of the girders as a result of the impact.

For this purpose, the TLS technology was selected on the basis that it offered the possibility of determining the entire structure’s geometrical deformations. The WD-113 object was scanned from 26 positions to obtain complete spatial information regarding the structure. The accuracy was assessed as typical of this technology, amounting to approximately 1 cm, which the girder analysis confirmed (whereby the error in the structure itself had to take into account a certain margin relating to construction errors in the constructed object). The overall point model of the structure was obtained as a result of the measurement and is shown in Figure 4. By analysing the deformations resulting from the impact, the theoretical line was modelled, designating its origin at the junction between the girder and the abutment. In order to determine the cross-section in the above-mentioned manner, the distance between it and the recorded point cloud was calculated. The results presented in Figure 5 provided an answer regarding the changes caused by the impact (the coordinate system in Figure 5 is identical to that in Figure 4A. The geometric results obtained were implemented for numerical calculations, the methodology of which is presented in the following subsections.

#### 2.3.1. Numerical Modelling of the Structure

**Figure 6:**
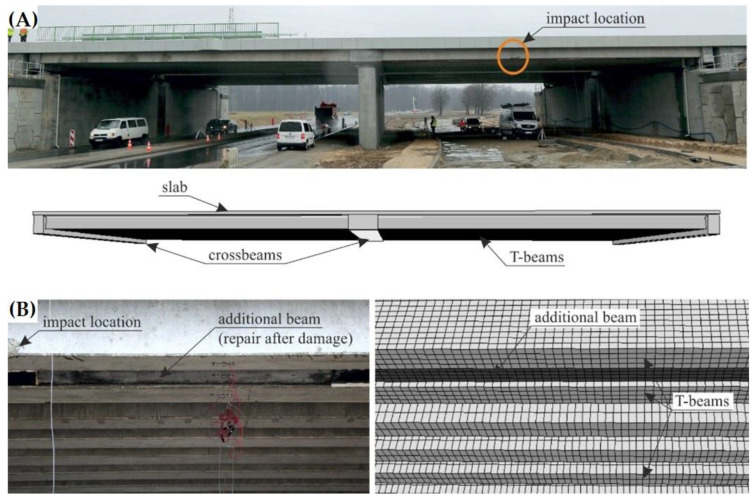
Comparison of the numerical model and the actual structure: (**A**) general view (the view of the FEM mesh view is turned off for better visibility), (**B**) view of the extreme T-beams and the additional beam made as a repair after the vehicle impact (the FEM mesh is visible).

The bridge structure’s numerical model was developed using the numerical model, which had previously been implemented in research [46]. The original model was developed to analyse the vehicle’s impact with the span of the bridge. Given that the present study concerns the same bridge structure, the model has been modified and adjusted for the purpose of this analysis. The numerical model takes the superstructure’s main elements into account—namely, the T-beams, the crossbeams, and the slab. The model also includes an additional concrete beam placed between the two end T beams. That additional beam was proposed and constructed as part of the repairs after the bridge had been damaged by the vehicle’s impact, as described in Section 2.1 and Section 2.2 Figure 6 illustrates the comparison between the developed numerical model and the full-scale WD-113P object.

**Figure 7:**
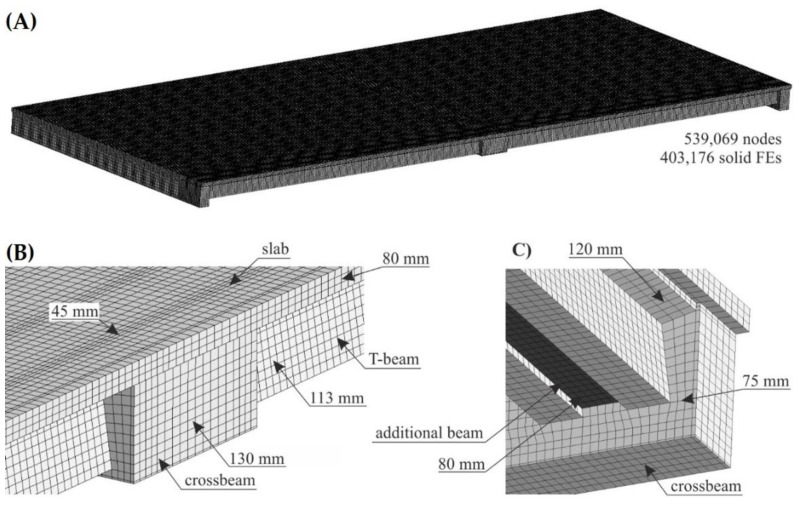
Details of the discretisation of the bridge structure numerical model: (**A**) general view of FEM mesh, (**B**) detail of the model in the vicinity of the intermediate crossbeam, and (**C**) detail of the model in the vicinity of the end crossbeam.

The bridge structure model considers only the concrete elements, which were modelled using 8-node constant stress solid finite elements (FEs). The numerical mode consists of 539,069 nodes and 403,176 FEs. Figure 7 presents the numerical model’s details. The dimensions of the side of the solid FEs ranged from ~40 to ~200 mm (see Figure 7B,C); for comparison, the bridge is 48.90 m long and 18.95 m in width. The concrete parts were assigned a linear elastic material model. The T-beams and the additional strengthening beam were made of C35/45 concrete, whereas the crossbeams and slab were made of C30/37 concrete. For the C30/37 concrete, the following parameters for the elastic material model were used: Young’s modulus was 32 GPa, and Poisson’s ratio was 0.2. For the C35/45 concrete, these values were 34 GPa and 0.2, respectively.

**Figure 8:**
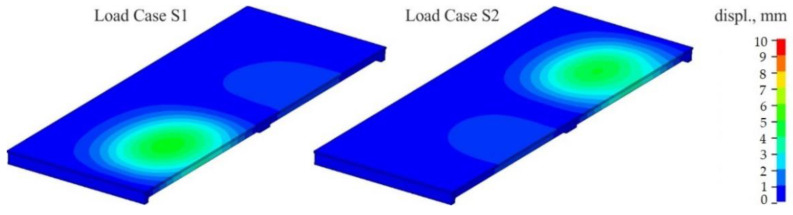
Resultant displacements for load cases S1 and S2.

The model considered two load cases: S1 and S2. In the S1 load case, the trucks were positioned in the span 1–2, while in the S2 load case, the trucks were in the span 2–3. In each load case, five trucks weighing 32 tonnes each were applied. In the numerical model, the concentrated nodal forces were applied to the appropriate nodes to reflect the position of the trucks and their wheels in the load cases. The values of those concentrated forces on each wheel were 30 or 50 kN. Given that each loading case involved the use of five 32 t trucks and that each truck had eight wheels, a total of 40 concentrated forces were applied in each load case. The structure’s supports were modelled in places of pot bearings. Axes nos. 1, 2 and 3 each have five supports (see Figure 1A), making a total of 15 supports. The supports of the two extreme cross-girders (i.e., in axes nos. 1 and 3) were modelled using simple supports for which the vertical translational degree of freedom (DoF) was fixed. However, for the middle supports in axes nos. 1 and 3; also, the displacement along the *y*-direction was constrained (see the coordinates in Figure 4A). The four supports for the intermediate cross-girder—two extreme ones on each bridge side—had a fixed translational DoF along axes *z* and *x*. The pinned support was used as the middle support in this axis no. 2. Linear static analysis was conducted, with the calculations performed using LS-DYNA MPP d R11.1.0 on the supercomputer Tryton managed by the Academic Computer Centre (CI TASK) in Gdansk, Poland. Figure 8 illustrates the structure’s displacements. The results will be discussed in greater detail in the sections that follow.

#### 2.3.2. The Scope of Tests and Instruments

**Figure 9:**
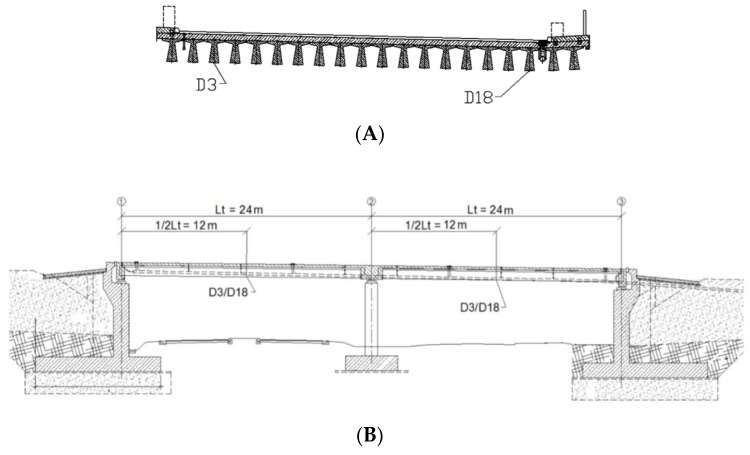
Arrangement of measuring sensors in cross-section (**A**) and longitudinal section (**B**) of the object.

In accordance with the Polish PN-S-10040: 1999 standard, the spans’ displacement was measured using sensors for measuring linear displacements. Figure 9 illustrates their respective arrangements in cross-section (A) and longitudinal section (B). The points were placed in the middle of the bridge span on the 3 and 18 “T” pre-stressed concrete beams, respectively. Lt denotes the span’s length, and D3/D18 denotes the numbers of the pre-tensioned concrete beams on which the sensor is mounted.

A Leica P30 scanner was used to obtain full spatial information about the measured object. This instrument provides 3D data at a speed of 1 million points per second within 270 m. A laser scanner was used for testing in view of the fact that the area that must be encompassed by measurements is a key challenge in construction. In the case of linear displacement sensors, it is possible to obtain information about displacements on one of the axes at one point only and—where total stations were used—in places where prisms were placed. The main drawback to using scanners compared to classic measuring equipment in the form of a total station is that they yield an accuracy of approximately 1–2 mm, while measurements taken using a total station allow for an accuracy of 0.5 mm. Therefore, the paper proposes that it may be possible to use laser scanning, sensors for linear displacements, or total stations and to interpret the results in tandem. The issue of the aggregational use and interpretation of data is widely documented in the literature. Solutions have been offered in practical (e.g., combining models derived from a laser scanner and high-resolution images for modelling ship hulls shown in reference [47]) or mathematical (in the case of proposals to use algorithms to combine two data sets, shown in publications [48,49]) terms. However, this research focuses on the joint use of spatial information from various sensors to provide comprehensive spatial information that pertains directly to displacements or deformations.

**Figure 10:**
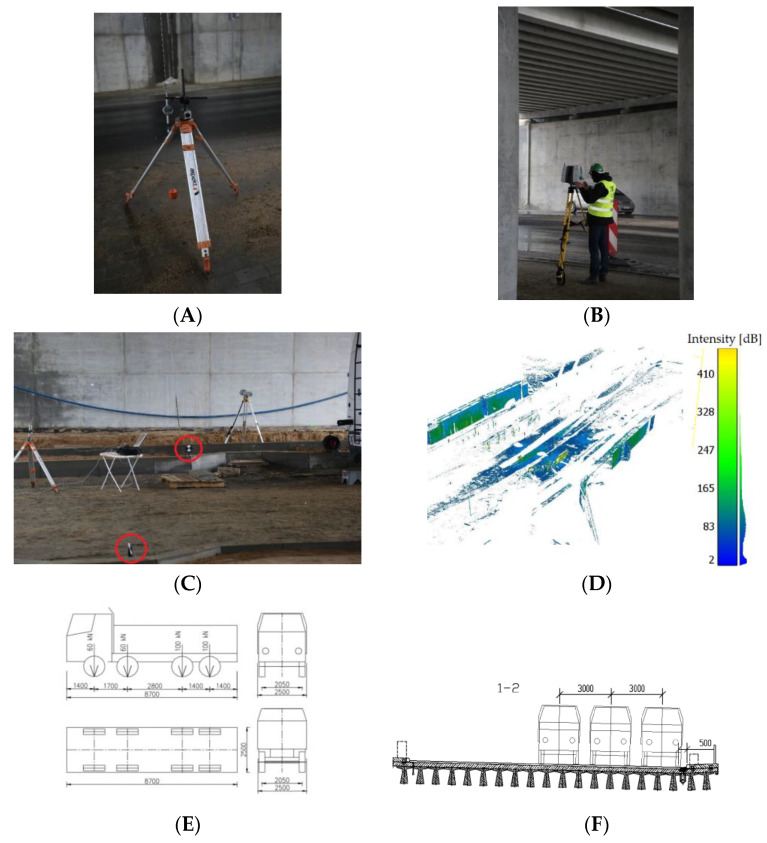
(**A**) Measuring sensor for measuring linear displacements; (**B**) a laser scanner when working with the operator; (**C**) TLS targets; (**D**) a separated point cloud applied to align data between scan positions; (**E**) pressure, wheel tracks, and dimensions of trucks loaded up to the calculation version; (**F**) view of the actual setting.

**Figure 11:**
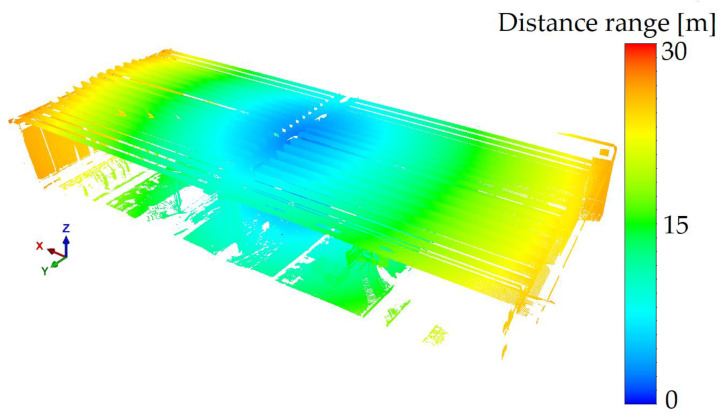
Distance range between scanner and concrete bridge.

The assumption that allowed us to obtain full spatial information regarding the object under examination was that two laser scanner stations were located under each of the bridge spans for each load setting. The scan positions were aligned to evenly spaced aiming targets, and the entire process was supported by the iterative closest points (ICP) algorithm, previously excluding from the set points in space characterised by a settled fear of displacement associated with the settlement of the bridge. The points included in the alignment were checked using precise levelling on each of the bridge columns supporting the structures. Figure 10 illustrates the measuring equipment used. Four-axle trucks (e.g., Mercedes 3538, Scania P124, MAN TGA) with a total weight of 32 t were accepted as the test load. The trucks were positioned first on the S1 load and subsequently on the S2 load. Additionally, in Figure 11, we have shown the distances between the scanner and the bridge girders. The centre of the span was approx. 15 m from the scanner, which (in the case of the manufacturer’s warranty) made it possible to measure with approx. 1.5 mm accuracy.

#### 2.3.3. Post-Processing of the Data

The post-processed data represent the results of the scheme shown in Figure 11.

**Figure 12:**
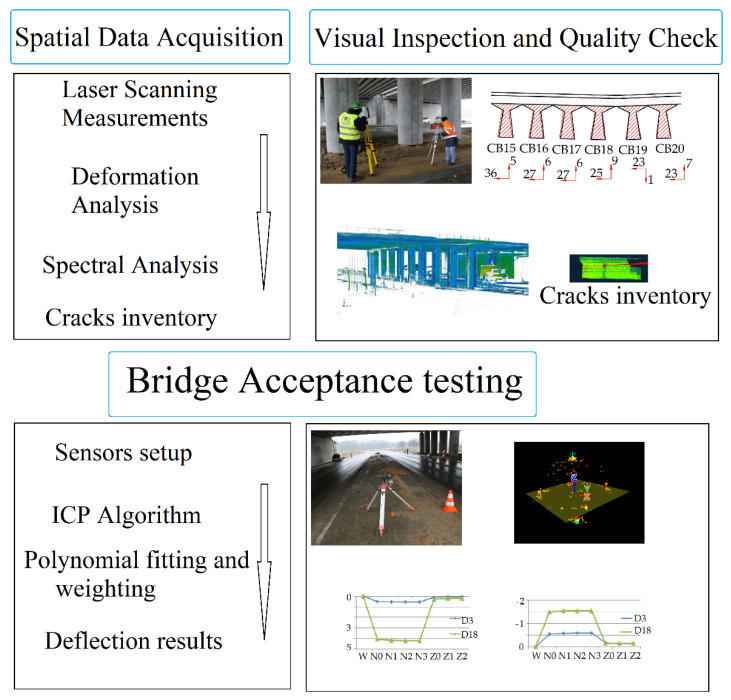
Processing methodology.

As Figure 12 illustrates, the process was divided into three stages. During the first stage, a visual inspection and applied additional measurements were performed to estimate the object’s technical condition. The results obtained facilitated the development of the FEM design methodology and the selection of an appropriate measurement method. The chosen method, which includes laser scanning measurements and potentiometric sensor setup, does not require time-consuming sensor mounting, and the results obtained will be optimal for use permit statements.

**Figure 13:**
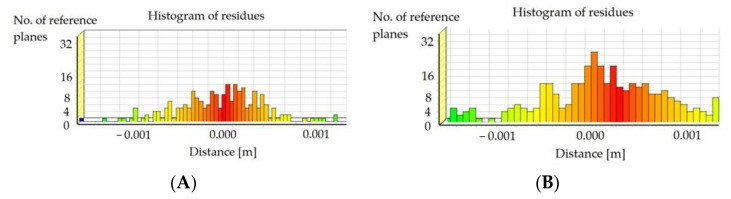
Results of scan position alignment between (**A**) reference and S1 and (**B**) reference and S2.

After selecting the laser scanning method and mounting the potentiometric sensors, the necessary data were collected to confirm the design’s compliance with the methodology described. The measurement data were developed in three stages. First, the point cloud was aligned between the two positions. Next, the reference plane was fitted, and the cross-section for analyses was marked out. This cross-section had to pass through the mounting points of the potentiometric sensors. The final step was to determine the differences in the altitude coordinates of the scans for two load schemes and to fit the polynomial function that approximated the results. Based on the statistical values obtained, the best fit of the cloud was matched with the use of sensors. In this way, it was possible to obtain polynomial reference values on the basis of numerical calculations. The histograms of the point cloud alignments estimating the mean errors of this connection are shown in Figure 13. Refining the results, Figure 13A shows the alignment of the reference state measurements to load S1 with respect to one another, and Figure 13B illustrates the alignment of the reference state measurements to load S2.

In order to obtain optimal results up to an accuracy of 1 mm (within the error of the measurement itself), an automatic plane search was applied using the ICP algorithm (the so-called Plane Patch Filter). An appropriate cross-section was selected using a simple mathematical analysis. Using the least squares method, the plane passing through the installed potentiometric sensor points was fitted, and points were marked at a distance of up to 5 mm from this plane in two directions, resulting in a section thickness of 1 cm. Next, a noise filter was used to locally fit the planes at several points and remove points that generated a too-large polynomial fitting error. The remaining noise was manually removed during the visual inspection of the obtained result.

Having determined the appropriate cross-sections, we analysed the deformations that occurred as a result of the load as follows: by defining the reference FEM model, it was possible to achieve a functional approximation of the results showing the deflection of the span. Polynomial fitting is a well-established and widely applied method; therefore, if certain rules are observed, it can be applied to laser scanning, thus enhancing the certainty of the results. In order to achieve this, the statistical values of the functions were first assessed from the data yielded by the FEM model. When data are obtained from scanning in accordance with traditional measurement techniques, the deflection tendency should be similar to that obtained using FEM calculation. Next, for points located in the locations of the potentiometric sensors, the heights of the scanner points were changed to align with these sensors. The measurement data that were modified in this way were adjusted until a correlation with the statistical parameters of the FEM model was obtained. The results obtained in this way constituted a reference for the analysed building’s serviceability state. Significantly, the statistical values obtained in this way were presented in millimetre form in the approximation of the measurement points to the function (residuals norm) and the values of the deflections. To search for correlation, we simply segmented the cross-section for each of the girders and adjusted the weights of the deflection values, reducing them in accordance with the increase in distance from the potentiometer sensor. We estimated the weight-reduction threshold by a step of 0.1.

## 3. Experiment Results—Comparison of Measurements

After visually assessing the structure, we approved the calculation model and selected test methods that would reliably check the structural response. Therefore, it was decided to apply the original solution using a laser scanner to examine the engineering structure. This relates to the aggregated use of sensors for linear displacement, which enhances analytical precision. Laser scanners are capable of observing entire structures and assessing their conditions. The use of low-cost solutions in the form of sensors to measure linear displacements can help enhance the results’ reliability and allow the FEM model to be verified. Based on the alignment of the data, we applied the ICP algorithm [48,49,50,51,52] and evenly distributed the targets, which were treated as references [53,54,55]. Given that the bridge structure consisted of two spans, the scanner position was not at the same point during the tests. Therefore, the coordinates transformation consisted of calculating the data from two scan positions into a local coordinate system. An additional problem relating to the scan positions’ alignment was noticed: the points for the ICP algorithm could not be used in places of deflection. Therefore, precise levelling measurements were performed on the structure’s columns and abutments. Because they did not deform, we appropriately limited the range of point clouds by subtracting the upper structural part. Finally, we evaluated the results obtained and compared them to those obtained at the design calculation stage. Taking the publications [56] into account, the scan results should not differ significantly from those obtained at the numerical calculation stage.

### Comparison of Measurements

To properly assess the deformation results and summarise the theses proposed at the beginning of this work, it was decided to present the results as follows:

(a) Numerical tests,
(b) Laser scanning without translation imposed by linear displacement sensors, and
(c) Laser scanning with translation imposed by linear displacement sensors.

**Figure 14:**
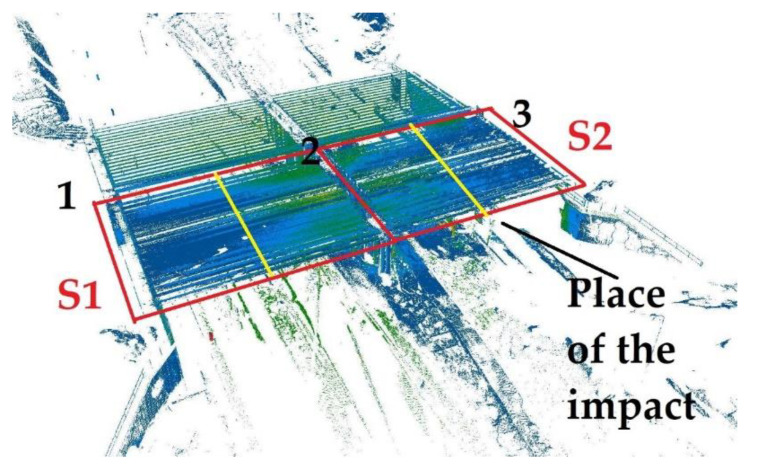
The sections used to compare the results for the S1 and S2 loads in the middle of each span are marked in yellow.

**Figure 15:**
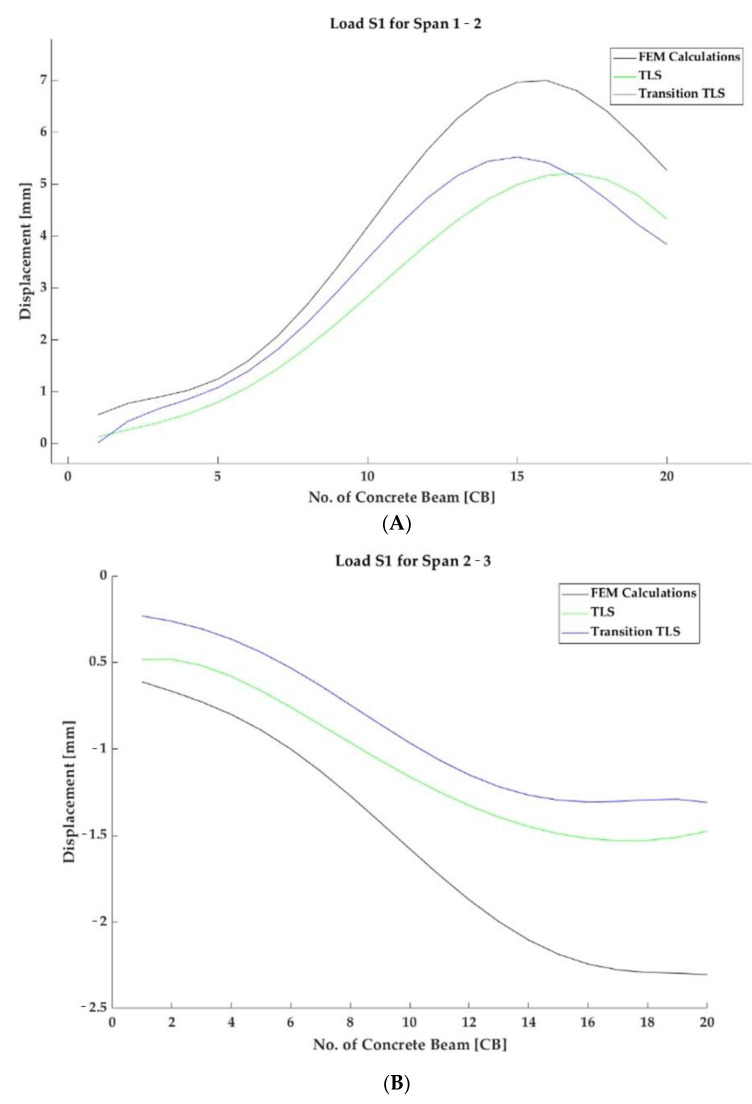

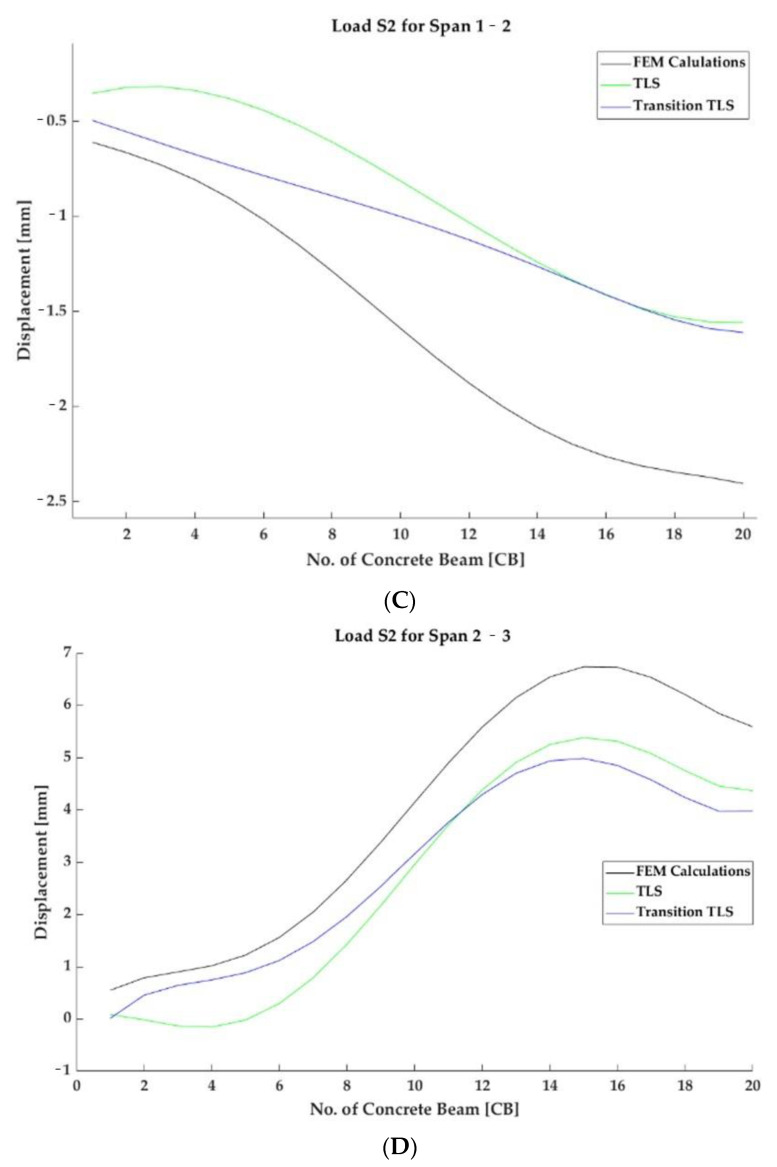
Displacement results for two loads in the cross-section for each span shown for numerical calculations, a laser scanner, and a scanner aggregated with potentiometric sensors. The symbols denote (**A**) S1 load for the 1–2 span, (**B**) S1 load for the 2–3 span, (**C**) S2 load for the 1–2 span, and (**D**) S2 load for the 2–3 span.

Figure 14 presents the results as a selected cross-section for each span, marked S1 and S2, respectively. Figure 15 presents a comparison of the results. The sections were selected due to the fact that the numerical tests were performed just at the midpoint of each of the spans and that sensors for linear displacement measurements were installed in these places, ensuring their reliable results in accordance with traditional approaches to testing bridge acceptance. As a reminder of the scope of the research, theoretical deformations of the structure in the middle of the span were calculated from the numerical tests, and this result, in reference to previous works, yielded a result similar to the actual one. Where a reflectorless measurement was taken with a laser scanner, the theoretical accuracy of the obtained results was approximately 2 mm at 10 m, and a more precise result may be expected when the point cloud is translated.

The most accurate measurements verifying the structure’s deformation under load are provided by sensors used to measure linear displacements placed under the prefabricated concrete element and marked as 3 and 18. These measurements are the most accurate because they are capable of showing deformation up to 0.1 mm. Table 1 synthesises these results.

## 4. Discussion

The discussion begins with an estimation of the costs and workload for the experiment in comparison to acceptance tests typically performed on bridge structures. First, a computational model made by an accredited laboratory is accepted. Next, the measuring points on the tested object are estimated, and a measurement technique is selected. This solution generates several logistical problems. In order to mount points on the construction, their number would need to reach several dozen to ensure a result that was sufficiently reliable for comparison with the numerical model. The assembly of these points is time-consuming. Moreover, each must be appropriately adapted to the instrument’s position and dismantled after the task has been completed. Furthermore, each measurement epoch may be expected to last several minutes unless multiple total stations are used, in which case, the time taken will be reduced, but equipment and personnel costs will be higher. In the case of laser scanning technology, the measurement time was significantly reduced (one scan lasted around two minutes), and the targets were on the ground, which supported the alignment process. Their placement on the structure is brief compared to the prisms from a total station. In the research, one drawback was observed to this solution, which is also evident from the graphs illustrated in Figure 15. It concerns the obtained accuracy, which, in comparison with the reference potentiometric sensors, reaches up to 2 mm. The measurement of 2 mm refers to the accuracy of the laser scanning technology while two scan positions are aligned with each other. In the cross-section (marked in yellow in Figure 13), where the deformation curve was indicated by numerical calculations, we noticed that the scanning technology might be used as a “replacement” for standard measurement techniques by virtue of its speed of measurement and the universality of the data obtained. Significant possibilities have arisen as a result of the development of 3D models and their subsequent evolution [57] and the technical advantages of using the devices themselves [58], and their subsequent development [59,60,61]. In view of the above, it is worth mentioning other aspects that relate to the legitimacy of the research’s originality. Therefore, we propose to continue the research in terms of standardising the results of the acceptance tests. Laser scanning itself provides a continuous dot model similar to that obtained by numerical methods (see Figure 7 and Figure 14). This means that knowing the reference shape of the function, and we can first evaluate it in terms of its polynomial character during static loads. Noting the acquired dependence, we concluded that it is justifiable to conduct research to verify whether it is possible to use the technology in the acceptance process and to perform calculations on the point cloud when analysing the structure’s load patterns.

By obtaining the appropriate function of the structure’s deformation at the midpoint of each span, we were able to transform it using two reference points from the potentiometric sensors. They were marked as the number of prefabricated elements and indicated their deformation along the height coordinate. It appears that, during these assumptions, the result may be used as a certainty with each subsequent test. Therefore, we may speak of certainty of measurement within a certain tolerance. We defined this tolerance as the value of the noise generated on the prefabricated elements tested. With a deformation size of several mm maximum, the surface of each prefabricated element becomes instrumental in the obtained result. When losses emerge, or the beam shows a relatively large angle of incidence, the points recorded may distort the result during spatial analysis. Furthermore, when a polynomial is fitted, cross-sectional analysis can easily remove outliers. Therefore, we performed the appropriate point cloud filtration, fitting the surface to each of the prefabricated elements and then removing the points that protruded from it by a value above double the fit error (i.e., above the absolute value of 4 mm from these surfaces).

It is evident that some points (particularly those on the edges of the prefabricated element) were registered as outliers. It would be erroneous to determine deformations as having occurred in these places. In addition, it is not possible to use the points from the potentiometers to show the entire structure, as we dealt with the deformation diagrams in the middle of the span. Potentiometers would thus have to be placed in greater numbers. In our opinion, the best solution would be to place them on every second pre-fab, which would generate as much work as would be required to measure using standard measurement techniques. Numerically calculated deformations refer to the centre of the span of each span in the two load patterns, S1 and S2. During fieldwork, it is important to verify that the calculation values are correctly observed in the structure. Owing to the bridge’s construction, the use of standard measurement methods would be costly and time-consuming compared to laser scanning. In the case of deformation values of approximately 1 mm, the scanner is capable of showing them all, owing to operations on the function specified during numerical calculations. However, taking into account the technological possibilities currently available, we wish to understand how the entire structure works under load. In this case, however, the obtained results should also be correctly interpreted. They show deformations under the influence of loads, but some values are greater than those measured using deformation-measuring devices. Following closer examination, it emerged that they mostly represent the noise generated by the construction itself and the angle of the scanner beam on it. Therefore, we estimate that for inventory applications, the data may be reused; however, to investigate the places marked in red, the number of positions should be increased, and points where the angle of incidence is greater than the set value, should be discarded. Therefore, for the analysis, we recommend that outliers be rejected or that the possibility that the noise phenomenon can be analysed by accessing the point cloud and photographic documentation.

The results discussed must be compared with the research in Section 2.2, in which the methodology presented in this article has not been used. The unaccredited tests, which were used, appeared to comply with the conditions of bridge test loads. Unfortunately, after an in-depth analysis, it turned out that on the map deformation of the scan, there are places that do not correspond to the readings from displacement measuring sensors (we assumed that the analysis is based on differences greater than 20% of the total deflection value). There are several factors influencing this state of affairs: continuity of points, surface fit error, and errors related to 3D modelling. In this way, we showed that from a Civil Engineering perspective, a laser scanner could be a good non-accredited solution supporting basic bridge research. In our methodology, the indication points of the potentiometric sensors are not directly implemented. Depending on the distance of the point cloud model from these sensors, the values of deflections are weighted, thus obtaining a deflection graph consistent with the actual state of the structure.

Further research relating to the proposed methodology’s development will be related to the acquisition of additional spectral information on the structure. As noted in the introduction, in light of the changing atmospheric, lighting and logistic conditions (in terms of the arrangement of, e.g., prisms or additional sensors), laser scanning theoretically fulfilled its role. However, conclusions may be drawn from the observation of multiple points in the cross-section on which the vertical displacement sensors are placed. This creates a situation in which our method has a certain limitation. In connection with the above, it is necessary to propose a methodology for determining the behaviour of the entire span of the structure without approximation of points located on it while reducing costs and minimising the time needed for research. In our opinion, the appropriate solution may not be to use additional numerical calculations but to add other sensors (e.g., in the form of high-resolution multispectral recording) to create span families in BIM (Building Information Modelling) technology. The subject’s metadata may include the results of numerical calculations, the registered wave spectrum, or additional numerical tests (e.g., using the DIC method). Such technology is critical for efficient infrastructure management.

## 5. Conclusions

We achieved our goal of performing non-standard measurements on a bridge structure using a laser scanner. The methods used allowed us to comply with the actual behaviour of the structure with the previously performed FEM simulations. Moreover, the main advantages of our method are as follows:

- We are able to observe all points on the structure in a selected cross-section during the test.
- The laser scanning technology operates irrespective of lighting conditions and is resistant to weather conditions.
- The tests verified that 0.5 mm accuracy was obtained using the method described herein.
- The test confirmed the design response in accordance with the FEM calculations’ predictions, and the object was approved for use.
- Future research should be related to the computerisation of monitoring solutions in BIM technology and the use of additional sensory solutions.

To achieve these conclusions, we performed the following steps: the first solution was to adapt the precision requirements to the numerical project. For this purpose, we used potentiometric sensors to measure linear deformations. Then, we performed laser scanning measurements using two scan positions. Finally, we filtered the data for the selected cross-section and modelled points into polynomial deflection. We anticipate that our methodology will be applicable to the confirmation of numerical calculations on other structures [62] or bridges, which form the core of our team’s work [63].

## Figures and Tables

**Table 1:** Measurement results during loading.

	Span 1–2						Span 2–3					
No.	CB 3			CB 18			CB 3			CB 18		
	[mm]			[mm]			[mm]			[mm]		
	Linear	FEM	TLS	Linear	FEM	TLS	Linear	FEM	TLS	Linear	FEM	TLS
S1	0.80	0.90	0.50	4.64	6.40	4.50	−0.33	−0.70	0	−1.30	−2.30	−1.50
S2	−0.59	−0.70	−0.50	−1.55	−2.30	−1.00	0.54	0.90	0	4.21	6.10	4.00

## Data Availability

Not applicable.
